# Shared and Unique Patterns of Dysregulated Dual Systems Between Adolescent Problematic Video Gaming and ADHD

**DOI:** 10.1002/pchj.70113

**Published:** 2026-07-13

**Authors:** Ruijuan Wu, Kunru Song, Yishan Shen, Jintao Zhang, Yueqin Hu

**Affiliations:** ^1^ Faculty of Psychology Beijing Normal University Beijing China; ^2^ Faculty of Education University of Macau Macua SAR China; ^3^ School of Family & Consumer Sciences Texas State University San Marcos USA; ^4^ State Key Laboratory of Cognitive Neuroscience and Learning and IDG/McGovern Institute for Brain Research Beijing Normal University Beijing China

**Keywords:** ADHD, dual‐systems, inhibitory control, problematic video gaming, reward sensitivity, transdiagnostic

## Abstract

The association between problematic video gaming (PVG) and ADHD has long attracted attention. Dual‐systems models posit that reward sensitivity (RS, the tendency to pursue rewards), inhibitory control (IC, the capacity to suppress prepotent responses), and the imbalance between them (where one system predominates over the other) constitute a useful framework for explaining their association. However, empirical tests of whether and how specific dual‐system mechanisms explain the association or phenotypic differentiation of PVG and ADHD remain limited. We hypothesize that (H1) both RS and IC predict PVG and ADHD symptoms; (H2) the degree of imbalance between RS and IC is associated with both PVG and ADHD. Additionally, we explore whether the specific associations between imbalance and symptoms differ between PVG and ADHD. Two waves of data from 10,973 participants (52.43% male, aged 12–14 years) from the Adolescent Brain Cognitive Development (ABCD) study were analyzed. Cross‐lagged panel models (CLPM) and Response Surface Analysis (RSA) were used to assess shared and unique relationships of RS, IC, and their imbalance with PVG and ADHD. CLPM analyses found that RS predicted subsequent IC, IC predicted subsequent ADHD, and ADHD predicted subsequent PVG, suggesting H1 was only partially supported. RSA revealed that a greater degree of imbalance was associated with more severe PVG and ADHD symptoms (supporting H2), and that imbalance configurations exhibited both shared and disorder‐specific patterns between PVG and ADHD. Specifically, reward sensitivity showed a linear association with PVG but a curvilinear association with ADHD, and IC correlated with ADHD only. We observed similarities between PVG and ADHD in the degree of dual‐systems imbalance, alongside differences in imbalance configuration. These findings provide theoretical insight into dual‐systems features linking PVG and ADHD and may inform future research on early identification and prevention in high‐risk populations.

## 1 Introduction

Problematic video gaming (PVG) and attention‐deficit/hyperactivity disorder (ADHD) are two prevalent behavioral problems in adolescents (Koncz et al. 2023). The global prevalence rate of PVG is estimated at 6.04% (Meng et al. 2022), and that of ADHD ranges from 10% to 13% (Centers for Disease Control and Prevention 2024), which represent significant public health concerns. PVG is defined as a behavioral pattern characterized by impaired control over gaming, prioritization of gaming over other interests, and continued engagement despite negative consequences (Tejeiro Salguero and Morán 2002). ADHD is a prototypical behavioral disorder characterized by reduced attention span, hyperactivity, and impulsivity (Hechtman 2016). Although a growing body of research has reported statistical associations between PVG and ADHD, the underlying mechanisms remain unclear (Kim et al. 2017; Karaca et al. 2017). Some unidirectional hypotheses suggest that PVG may result from ADHD. For example, Han et al. (2009) posited that individuals with ADHD might use gaming as a coping strategy to alleviate symptoms. However, emerging evidence suggests a possible shared mechanism between PVG and ADHD, that is, the dysregulation in the reward and inhibitory system (Grassi et al. 2024; Koncz et al. 2023). Etiological theories of PVG (e.g., Dong and Potenza 2014) ADHD (e.g., Sonuga‐Barke 2003) also separately highlight heightened risk associated with altered reward‐inhibition interactions. Yet, direct empirical investigation into such claimed mechanisms remains scarce (Scheres et al. 2024). Thus, comparative studies incorporating PVG and ADHD with reward and inhibitory systems are necessary.

### 1.1 The Development of Dual Systems in Adolescence

Theoretically, the reward and inhibitory systems have already been incorporated into a dual‐system framework, which is increasingly becoming a popular perspective for understanding adolescent problematic behaviors, such as PVG and ADHD (Lindgren et al. 2019; Wasserman et al. 2017). It distinguishes a bottom‐up reward system that drives reward‐seeking and impulsive behaviors (e.g., excessive video gaming) and a top‐down inhibitory control system that deliberately regulates one's behaviors to avoid negative consequences (Shulman et al. 2016). The two systems are theoretically treated as two distinct yet interacting roots of PVG and ADHD. In spite of inconsistent terminology in the existing literature (Evans and Stanovich 2013), we followed Shulman et al. (2016) and termed it the Dual Systems Model in this study.

The central tenet of the Dual Systems Model posits that the functional imbalance between the reward and inhibitory systems may serve as one of the etiological factors for problematic behaviors. Specifically, this model characterizes the imbalance as a developmental asynchrony during adolescence, where the reward system matures rapidly while the inhibitory system develops more gradually, producing a temporary bias toward reward‐oriented behavior without sufficient inhibitory controls (Casey et al. 2008; Luna and Wright 2016; Steinberg 2008). Such an imbalance is widely invoked to explain adolescents' elevated propensity for maladaptive outcomes relative to childhood and adulthood, including heightened risks for PVG and symptoms related to ADHD (Li et al. 2020; Thorell 2007). To operationalize these systems at the behavioral level and thereby link mental processes with real‐world practices, numerous proxy measures were proposed. For example, self‐reported reward sensitivity, sensation seeking, and intertemporal choice task‐derived delay discounting have been used as indicators of the functional level of the reward system (Persson et al. 2020; Zhou et al. 2022). In parallel, inhibitory control, cognitive flexibility, and self‐control ability have also been adopted in several studies to reflect the inhibitory system function (Duell et al. 2016; Shiels and Hawk 2010). In this study, we followed the updated recommendations from Shulman et al. (2016) that focus on reward sensitivity (RS) and inhibitory control (IC) as behavioral measures for the Dual Systems Model, in which the two constructs are assumed to widely map onto the core function of the reward and inhibitory systems, respectively (Adisetiyo and Gray 2017; Shulman et al. 2016).

### 1.2 The Associations Between Dual Systems and PVG/ADHD

Significant symptom‐level associations between PVG and ADHD have been repeatedly reported. A recent meta‐analysis synthesized this evidence and showed a moderate correlation between PVG severity and ADHD symptoms (meta‐analytic *r* = 0.296), which constitutes the motivation for investigating the potential shared mechanism (Koncz et al. 2023). The Cognitive‐Behavioral Model (Dong and Potenza 2014) and the Tripartite Neurocognitive Model (Wei et al. 2017) already emphasize the important role of reward system hypersensitivity (high RS) and inhibitory control deficits (low IC) in the emergence of PVG, which resonates with the Dual Systems Model. However, existing data primarily focus on isolated effects of RS and IC. For example, two longitudinal structural equation model studies showed that earlier IC deficits statistically predicted subsequent PVG (Jeong et al. 2019; Liu et al. 2025). Another study using linear mixed modeling reported a significant interaction effect between neuroimaging‐derived RS measures and measurement time in association with PVG, suggesting bidirectional relations between PVG and RS (He et al. 2025). There are also similar research practices in the field of ADHD studies. For example, case–control studies reported accelerated physiological responses to reward (a proxy of high RS) in children with ADHD (Luman et al. 2008), and reviews synthesized links between higher RS and more ADHD symptoms (Fairchild 2011; Luman et al. 2010). Meanwhile, intervention studies and reviews provided consistent evidence that stronger IC is associated with fewer ADHD symptoms (Sun et al. 2023). Longitudinal studies also indicated that changes in IC can predict subsequent changes in ADHD symptoms, whereas the reverse prediction is less often observed (Pang et al. 2025).

Although these studies provide a basis for testing dual‐system hypotheses, most investigations examine only one system in isolation, which often fails to consider the imbalance between RS and IC. In contrast, a subset of studies attempted to quantify such an imbalance using a single behavioral task. For example, based on the idea that the imbalance manifests as increased “acting without thinking” (Romer et al. 2017), Zhou et al. (2022) operationalized dual‐system imbalance via decision‐making tasks, treating imbalance as impairment primarily in one system (either heightened RS or reduced IC), and assessing it using monetary‐reward paradigms (e.g., the cup task). Although the single behavioral task can capture dynamic regulatory competition between the two systems, it is inherently time‐sensitive and may introduce substantial momentary bias, making it less suitable for addressing trait‐level questions. Furthermore, such task‐derived measures fail to uncover the unique contribution of each system, yielding only limited concordance with the Dual Systems Model (Persson et al. 2020). Accordingly, approaches that more stably capture individual differences in imbalance are needed. For example, imbalance indicators based on normative references and validated across multiple waves may provide a more reliable characterization of individual differences in dual‐system imbalance. Therefore, a combined perspective on the trait‐level imbalance is warranted, and this perspective should simultaneously examine the shared and unique contributions of dual systems to PVG and ADHD.

### 1.3 Heterogeneity of Dual‐System Imbalance in PVG and ADHD

Echoing the tenet of the Dual Systems Model, unraveling the shared and unique contributions of RS and IC to the two behavioral problems requires not only examining their isolated effects but also investigating how the imbalance between them is associated with PVG and/or ADHD. However, there are significant heterogeneities in this imbalance. As a heuristic theory, the Dual Systems Model typically summarizes normative features of adolescent development at the group level and therefore often struggles to account for individual differences (Pfeifer and Allen 2016; Van Den Bos and Eppinger 2016). Casey et al. (2008) argued that individual variation in RS may amplify the increase in behavioral problems during adolescence. Likewise, impaired IC has been identified as an important contributor to heightened adolescent vulnerability (Constantinidis and Luna 2019; Knoch et al. 2006). Together, these findings give rise to a testable theoretical conjecture: even when the degree of imbalance between the dual systems is similar, such imbalance may not represent a single, uniform pattern but instead may occur in multiple heterogeneous configurations. These configurations may help explain individual differences in problem behavior as well as differences in symptom expression.

**FIGURE 1:**
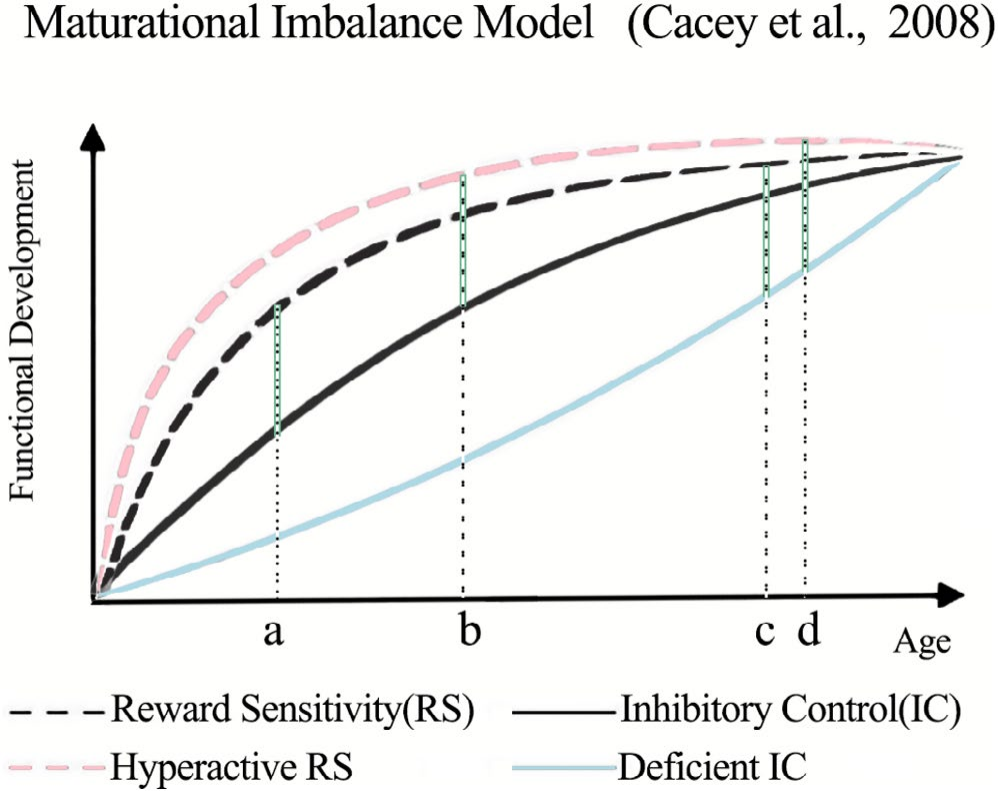
Dual systems model and diverse imbalance patterns. Adapted from Casey et al. (2008); Shulman et al. (2016), and Meisel et al. (2019). The figure depicts developmental trajectories of reward and control systems from late childhood to early adulthood. Pink and blue lines represent hyperactive RS and deficient IC, respectively, deviating from normative trajectories (black lines). Imbalance magnitude (vertical ΔRS‐IC) can manifest identically (e.g., green line at points a–d) yet reflect distinct maturity/abnormality configurations. Testing the imbalance hypothesis requires analyses that simultaneously quantify magnitude and pattern diversity.

As illustrated in Figure 1, even when the overall degree of imbalance is comparable, the specific combination of dual systems can vary (e.g., imbalance due to normative developmental timing; imbalance dominated by hyper‐reactive reward circuitry; imbalance driven primarily by weakened control processes; or imbalance produced by dysregulation in both systems). Whether these different configurations predict similar or distinct behavioral outcomes remains an open question. Prior studies modeling imbalance via interaction terms provide only rough insight into such heterogeneity. For example, research on substance use suggests that RS is associated with alcohol and cannabis use, particularly among adolescents with low IC (Peeters et al. 2017; Rhodes et al. 2013). Studies examining delinquent behavior have found that high RS prospectively predicts increases in delinquency only among adolescents with low levels of IC (Murray et al. 2021). Why is the interaction‐term approach coarse? Imagining two individuals with opposite profiles (e.g., RS *z* = +1 & IC *z* = −2 versus RS *z* = −1 & IC *z* = +2) can yield identical interaction scores despite potentially different outcomes. This limitation highlights the need for studies that simultaneously model different problem outcomes and use more refined methods to capture heterogeneity in dual‐system imbalance.

Regarding the heterogeneity of imbalance in PVG and ADHD, Adisetiyo and Gray (2017) proposed three testable hypotheses in a systematic review, including that the imbalance may manifest as co‐exaggerated deficits in both systems, reward‐specific impairments, or control‐specific deficiencies. Beyond the shared, problem‐general dual systems manifestations, these hypotheses indicate that problem‐specific manifestations of PVG and ADHD may arise from distinct configurations of the dual systems. Empirical comparisons highlight critical differences between addiction‐like problems (e.g., PVG and substance use disorder) and ADHD. For example, substance use disorder is primarily linked to different aspects of RS (e.g., sensation seeking and reward anticipation), whereas ADHD is predominantly linked to insufficient IC (Castellanos‐Ryan et al. 2014). Neuroimaging studies also found hyperactivation in RS‐related neurocircuits in substance use disorder and hypoactivation in IC‐related brain regions in ADHD (Van Ewijk et al. 2015). These findings imply that PVG may be more strongly associated with RS, while ADHD may be more closely tied to IC. However, direct comparisons are still lacking, and the exact picture remains to be elucidated.

### 1.4 Quantifying Dual‐Systems Imbalance via Response Surface Analysis

To sum up, two research gaps can be identified. First, the associations among RS, IC, and the two intercorrelated behavioral problems (i.e., PVG and ADHD) have not been rigorously tested together within a longitudinal design that establishes temporal ordering. Second, tests of the imbalance rarely simultaneously capture the dual systems, the degree of imbalance, and heterogeneity in imbalance configuration.

For the first gap, conventional multivariate longitudinal modeling can be useful (e.g., a four‐variable cross‐lagged panel model). However, the second gap requires a more refined analytic framework. Given this, response surface analysis (RSA) offers unique advantages for modeling three‐dimensional continuous relationships between two predictors and outcomes (Nestler et al. 2019). This method directly characterizes the joint effects of two continuous predictors on an outcome, as well as the continuous association between their discrepancy (e.g., when RS exceeds IC) and the outcome. It therefore allows researchers to simultaneously capture the independent contributions of RS, IC, and their imbalance, while also identifying the specific configurations of the two systems underlying imbalance. Accordingly, RSA can be a particularly suitable analytic strategy for testing imbalance‐based hypotheses.

### 1.5 Current Study

First, we examined the temporal associations among RS, IC, PVG, and ADHD using longitudinal models to address the lack of systematic longitudinal investigation in previous studies and to test potential etiological hypotheses. Then, we employed response surface analysis to examine the fine‐grained relationship between RS/IC and PVG/ADHD, by quantifying the degree and configuration of dual‐system imbalance, and thereby examining its associations with the risk states of PVG and ADHD. We hypothesize that: (H1) Both RS and IC predict subsequent PVG and ADHD symptoms. (H2) The degree of imbalance between RS and IC is associated with both PVG and ADHD. Additionally, we will explore an open research question regarding the specific configuration patterns of association between dual systems and symptoms, specifically in PVG and ADHD. Longitudinal data will be used to test H1 as a supplement to previous cross‐sectional studies, while concurrent nonlinear models will address H2 and the research question as an enhancement to previous linear investigations. Collectively, these endeavors will provide new insights and empirical evidence for the dual‐system hypothesis of PVG and ADHD.

## 2 Methods

### 2.1 Participants and Procedure

Participants in this study were drawn from the Adolescent Brain Cognitive Development (ABCD) Study, an ongoing 10‐year longitudinal investigation into youth mental health and neurocognitive development in the United States. This cohort includes 11,878 children, enrolled at ages 9 and 10 from 2016 to 2018, recruited from 21 sites nationwide through community outreach efforts. The sampling procedures were designed to ensure adequate diversity (see Garavan et al. 2018, for details). We utilized data from ABCD Data Release 5.1, where youth had completed their four‐year follow‐up assessment. The present study focused on the two‐year and four‐year follow‐up waves, as all variables of interest were completely measured at these waves. Given that PVG was first assessed at the two‐year follow‐up, we used data from this wave as the baseline values. Meanwhile, the dual‐systems variables (RS and IC) were not assessed at the three‐year follow‐up, but were assessed at the four‐year follow‐up wave. Therefore, the four‐year follow‐up wave was used as the earliest subsequent assessment with complete data on the key constructs. For clarity and simplicity, the two‐year follow‐up and the four‐year follow‐up are referred to as Wave 1 and Wave 2, respectively (see Table S1 for the original time labels and interview date ranges). Participants were excluded if they met any of the following criteria at either wave: (1) missing key demographic information (i.e., sex, age, race/ethnicity, parental educational attainment, or family income); (2) missing data on RS or IC; or (3) missing data on PVG or ADHD. A more detailed description of the ABCD data collection procedures can be found on the study website: https://abcdstudy.org/.

### 2.2 Measures

The measurement of variables in the ABCD study is described in previous publications (Barch et al. 2018; Weintraub et al. 2014). Here, we outline key measures utilized in this study.

*Reward Sensitivity* (RS) was measured by the BAS (Behavioral Activation System) subscale of the BIS/BAS scale (Carver and White 1994). The BAS subscale, comprising 12 items (e.g., “I feel excited and full of energy when I get something that I want.”), assesses three factors: Drive, Reward Responsiveness, and Fun Seeking. This measure has been widely used to assess individual differences in reward sensitivity. Respondents rated each item on a 4‐point Likert scale ranging from 0 (*not true*) to 3 (*very true*). Higher total scores (summed across 12 items) indicate greater RS. In the current sample, the Cronbach's alpha for the BAS subscale was 0.85 at Wave 1 and 0.86 at Wave 2.

*Inhibitory Control* (IC) was measured by the Flanker Inhibitory Control and Attention Test from the NIH Toolbox (Rueda et al. 2004). The task evaluates the ability to inhibit prepotent responses through 40 trials. Performance scores integrate accuracy and reaction time. Higher scores reflect better inhibitory control (Weintraub et al. 2014).

*Attention‐Deficit/Hyperactivity Disorder* (ADHD) symptoms were measured by the widely used and well‐validated Child Behavior Checklist (CBCL; Achenbach 2015). The CBCL includes 112 items in total, seven of which specifically measure ADHD (Cronbach's *α* = 0.84 at both waves), such as “Can't concentrate, can't pay attention for long.” Each item was rated on a 3‐point scale from 0 (*not true*) to 2 (*very true*). The total score was calculated by summing these items, with higher scores indicating more severe ADHD symptoms. This continuous indicator was used in the CLPM and RSA analyses. We also assessed and reported the prevalence in the current sample. ADHD‐positive case classification was determined via the K‐SADS‐5 interview (a computerized semi‐structured interview that assesses children's psychiatric conditions using DSM‐5 criteria). This categorical indicator was used to estimate prevalence.

*Problematic Video Gaming* (PVG) symptoms were assessed using the modified Video Game Addiction Questionnaire (VGAQ) (Andreassen et al. 2012), including six items (α= 0.85 at Wave 1; α= 0.87 at Wave 2) (e.g., “I spend a lot of time thinking about playing video games”). Responses ranged from 1 (*never*) to 6 (*very often*), and were summed to yield total scores, with higher scores indicating more severe PVG. This continuous indicator was used in the CLPM and RSA analyses. Similar to ADHD, we also report the prevalence of PVG using the VGAQ cut‐off (items scored > 3 were flagged; individuals with more than 3 flagged items were classified as PVG positive).

*Covariates* including age, sex, race/ethnicity, and the primary caregiver's highest educational attainment, as well as clinical variables such as major depressive disorder (MDD) and obsessive‐compulsive disorder (OCD), were modeled as time‐invariant terms. In contrast, combined family income was treated as a time‐varying covariate at each assessment wave (detailed in Table S3).

### 2.3 Statistical Analysis

First, to examine the longitudinal predictive relationships between RS/IC and PVG/ADHD, both the conventional cross‐lagged panel model (CLPM) and the random‐intercepts CLPM (RI‐CLPM) are methodologically possible. However, we used CLPM for two reasons. Methodological work warns that RI‐CLPM is ill‐suited to sparsely sampled, long‐interval designs because such data frequently lack the temporal density needed for stable and reliable estimation of the RI‐CLPM parameters (Asendorpf 2021). In addition, although RI‐CLPMs are valuable for isolating within‐person effects, they do not directly quantify how stable, between‐person differences propagate into later outcomes at the population level, which is a question central to our aims (Orth et al. 2021). Given our two‐wave structure and interest in population‐level cross‐lagged associations across a two‐year interval, the CLPM therefore provided the most appropriate framework for the present analyses. We thus employed CLPMs using Mplus 8.3.

We first constructed a bivariate CLPM including PVG and ADHD. Because theoretical accounts and prior empirical studies consistently report an association between these two behavioral problems, our first objective was to replicate that association in the current sample. Given the theoretical premise underlying shared dual‐system mechanisms, we anticipated they would demonstrate at least concurrent or even temporal relationships between them. Building upon this foundation, we then expanded the model to include RS and IC (a comprehensive CLPM), with a primary focus on examining whether RS and IC could prospectively predict future ADHD and PVG symptoms (i.e., the cross‐lagged paths). To control for potential reverse‐causality, we included longitudinal pathways from previous ADHD and PVG to current RS and IC. All analyses employed Full Information Maximum Likelihood (FIML) estimation to handle missing data.

Then, to investigate nuanced patterns of dual‐system imbalance in PVG and in ADHD, respectively, Response Surface Analysis (RSA) (Nestler et al. 2019) was employed using the following formula:

$$y_{i}=b_{0}+b_{1}x_{1i}+b_{2}x_{2i}+b_{3}x_{1i}^{\;2}+b_{4}x_{1i}x_{2i}+b_{5}x_{2i}^{\;2}+e_{i}$$

where $x_{1}$ represents RS, $x_{2}$ represents IC, and *y* represents PVG or ADHD. By integrating linear, quadratic, and interaction terms of RS and IC, this approach can simultaneously examine the associations between RS, IC, and PVG/ADHD (reflected by coefficients *b*_*1*_–*b*_*5*_). Additionally, RSA allows for the derivation of key parameters such as *b*_*1*_ *+ b*_*2*_ and *b*_*1*_ − *b*_*2*_, which represent the slopes of the response surface along the lines of congruence ($x_{1}=x_{2}$) and incongruence ($x_{1}=-x_{2}$), respectively. Given our focus on dual‐system imbalance, the response surface analysis of oppositional configurations (*b*_1_ − *b*_2_, representing the continuum from low RS/high IC to high RS/low IC profiles) held greater theoretical relevance than congruent configurations (*b*_1_ + *b*_2_, representing matched RS/IC levels ranging from both low to both high). All variables were standardized to *z*‐scores. Given the sample's adequate diversity, we used the sample mean as a normative‐development reference and indexed individuals' deviations from that mean to characterize differences on core predictors and outcomes. This approach yields a relatively stable measure of imbalance and facilitates direct comparability of results between PVG and ADHD. Analyses were conducted using the RSA package (Schönbrodt 2015) in R (version 4.3.1). Missing data were handled using listwise deletion, with results provided via multiple imputation (Table S4) and FIML (Table S5). While CLPM examined longitudinal relationships across waves, RSA examined nonlinear associations separately for Waves 1 and Wave 2.

### 2.4 Ethics

The ABCD study received Institutional Review Board (IRB) approval, and informed written consent was obtained from all participating sites during the data collection phase. The analyses conducted in the current study utilized deidentified data from the ABCD dataset, and as such, were deemed exempt by the Beijing Normal University IRB and Human Investigation Committee. This study complies with the principles outlined in the Declaration of Helsinki.

## 3 Results

Table 1 displays the demographic characteristics for the final analysis sample. A total of 10,973 participants [5753 females, age = 144.32 ± 8.03 months] were included in Wave 1 (2‐year follow‐up), and 4754 participants [2488 female, age = 168.98 ± 8.17 months] in Wave 2 (4‐year follow‐up). The sex ratio was comparable across the two waves. The sample comprised participants from diverse racial backgrounds. In Wave 1, 53.42% (*n* = 5861) were White, 14.23% (*n* = 1561) were Black, 19.72% (*n* = 2164) were Hispanic, 2.11% (*n* = 231) were Asian, and 10.53% (*n* = 1155) were Other. In Wave 2, the figures were similar, with 56.42% (*n* = 2682) White, 10.71% (*n* = 509) Black, 20.59% (*n* = 979) Hispanic, 2.31% (*n* = 110) Asian, and 9.97% (*n* = 474) Other. The prevalence of PVG was 10.89% (*n* = 877) in Wave 1% and 10.41% (*n* = 392) in Wave 2, while the prevalence of ADHD was 5.63% (*n* = 605) in Wave 1% and 4.80% (*n* = 220) in Wave 2. The two waves were demographically comparable, reducing the likelihood that demographic shifts explain findings, and both waves included a meaningful proportion of participants with PVG and ADHD, supporting subsequent analyses.

**TABLE 1:** Demographic characteristics and psychopathological profiles of the study sample.

	Wave 1 (*n* = 10,973)	Missing	Wave 2 (*n* = 4754)	Missing
	*M* (SD) or *n* (%)		*M* (SD) or *n* (%)	
Age (month)	144.32 (8.03)	1	168.98 (8.17)	0
Sex (males)	5753 (52.43%)	0	2488 (52.33%)	0
Family income	7.52 (2.32)	964	7.84 (2.124)	471
Parent education	18.62 (3.32)	82	18.73 (3.142)	70
Race‐white	5861 (53.42%)	1	2682 (56.42%)	0
Race‐black	1561 (14.23%)	1	509 (10.71%)	0
Race‐Hispanic	2164 (19.72%)	1	979 (20.59%)	0
Race‐Asian	231 (2.11%)	1	110 (2.31%)	0
Race‐Other	1155 (10.53%)	1	474 (9.97%)	0
MDD	41 (0.38%)	221	—	—
OCD	662 (6.17%)	243	—	—
ADHD	605 (5.63%)	223	220 (4.8%)	16
PVG	877 (10.89%)	2923	392 (10.41%)	989

Abbreviations: ADHD: ADHD positive cases via the KSADS‐5 interview (a computerized semi‐structured interview that assesses child psychiatric conditions using DSM‐5 criteria); MDD: Major depressive disorder; OCD: Obsessive‐compulsive disorder; PVG: PVG positive cases classified by the VGAQ cut‐off (items scored > 3 were flagged, individuals with more than 3 flagged items were classified as PVG positive); wave 1 = 2‐year follow‐up, wave 2 = 4‐year follow‐up.

### 3.1 Descriptive Statistics and Correlations

The descriptive statistics and correlations were presented in Table 2. ADHD was significantly correlated with RS (*r*_wave1_ = 0.105, *r*_wave2_ = 0.045, *ps* < 0.001) and IC (*r*_wave1_ = −0.086, *r*_wave2_ = −0.052, *ps* < 0.001). PVG was significantly correlated with RS (*r*_wave1_ = 0.205, *r*_wave2_ = 0.010, *ps* < 0.001) and IC at Wave 1 (*r*_wave1_ = −0.04, *p* < 0.001; *r*_wave2_ = 0.03, *p* = 0.17). The correlation between RS and IC (*r*_wave1_ = −0.02, *p* = 0.04; *r*_wave2_ = 0.03, *p* = 0.14) was also only significant at Wave 1 with a very small effect size. These correlations aligned with the theoretical claims grounded in the Dual Systems Model.

**TABLE 2:** Descriptive statistics and correlations of PVG/ADHD and RS/IC.

	*M* (SD)_wave 1_	*M* (SD)_wave 2_	PVG	ADHD	RS	IC
PVG	12.46 (6.34)	12.86 (6.243)	1	0.18***	0.10***	0.03
ADHD	2.3 (2.77)	2.02 (2.605)	0.20***	1	0.05***	−0.05***
RS	16.17 (6.26)	16.38 (6.06)	0.21***	0.10***	1	0.03
IC	100.1 (7.71)	104.3 (7.314)	−0.04***	−0.09***	−0.02*	1

*Note:* Correlation coefficients for Wave 1 (2‐year follow‐up) are shown in the lower triangular matrix, with Wave 2 (4‐year follow‐up) coefficients presented in the upper triangular matrix.

* *p* < 0.05.

*** *P* < 0.001.

### 3.2 Longitudinal Relationship Between PVG and ADHD

**FIGURE 2:**
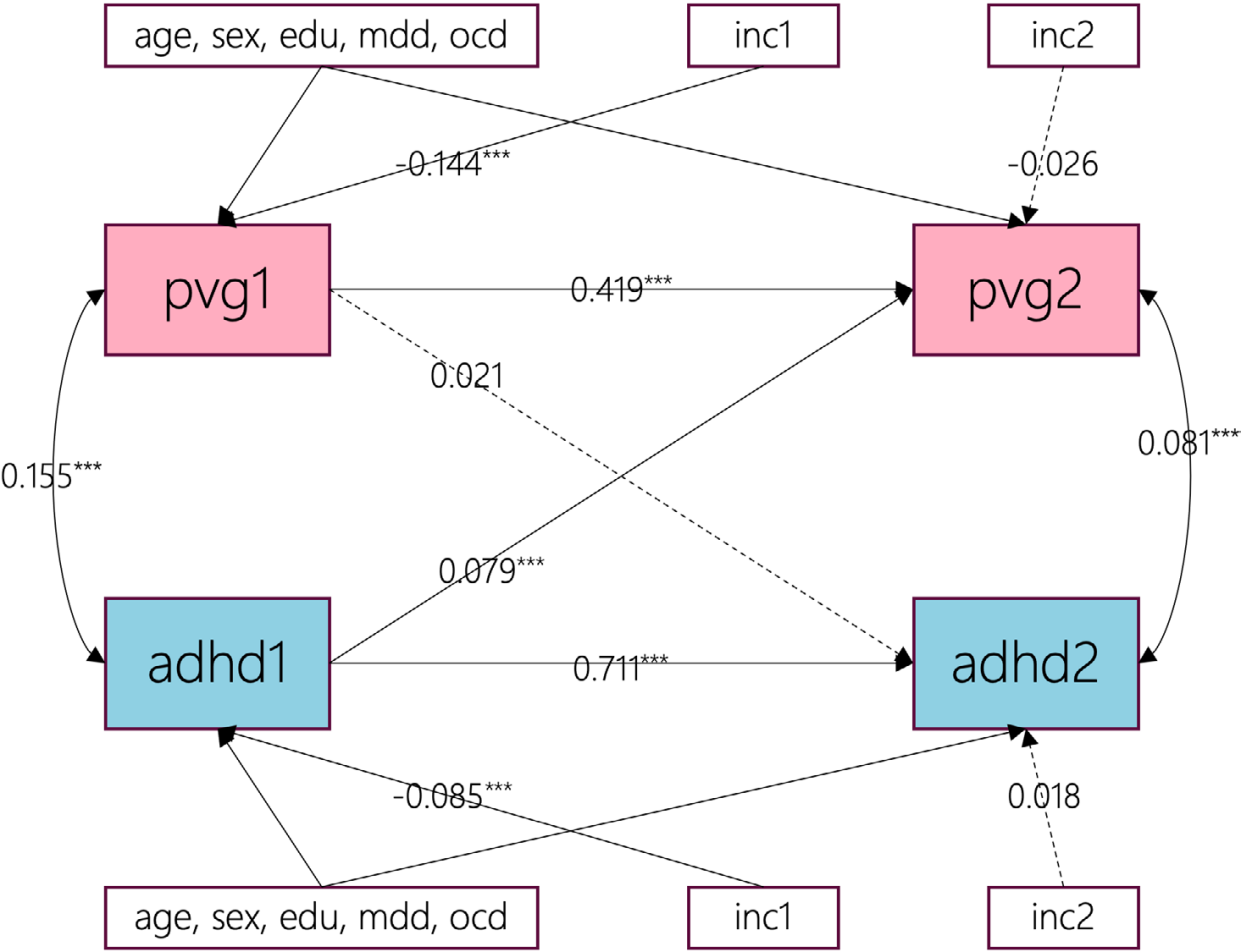
The Cross‐lagged panel model of PVG and ADHD. ***p < 0.001; inc: combined family income; edu: primary caregiver's highest educational level; mdd: major depressive disorder; ocd: obsessive‐compulsive disorder. For clarity of presentation, error terms and intercepts are omitted from the figure.

Figure 2 shows the basic bivariate CLPM of PVG and ADHD. There was a unidirectional relationship between PVG and ADHD. Specifically, ADHD symptoms significantly predicted subsequent PVG symptoms (*β* = 0.079, *p* < 0.001), but not vice versa (*β* = 0.021, *p* = 0.198). There was also a significant contemporaneous positive correlation between PVG and ADHD (*r*_wave1_ = 0.155, *r*_wave2_ = 0.081, *ps* < 0.001).

### 3.3 Longitudinal Relationship Between PVG, ADHD, RS, and IC

**FIGURE 3:**
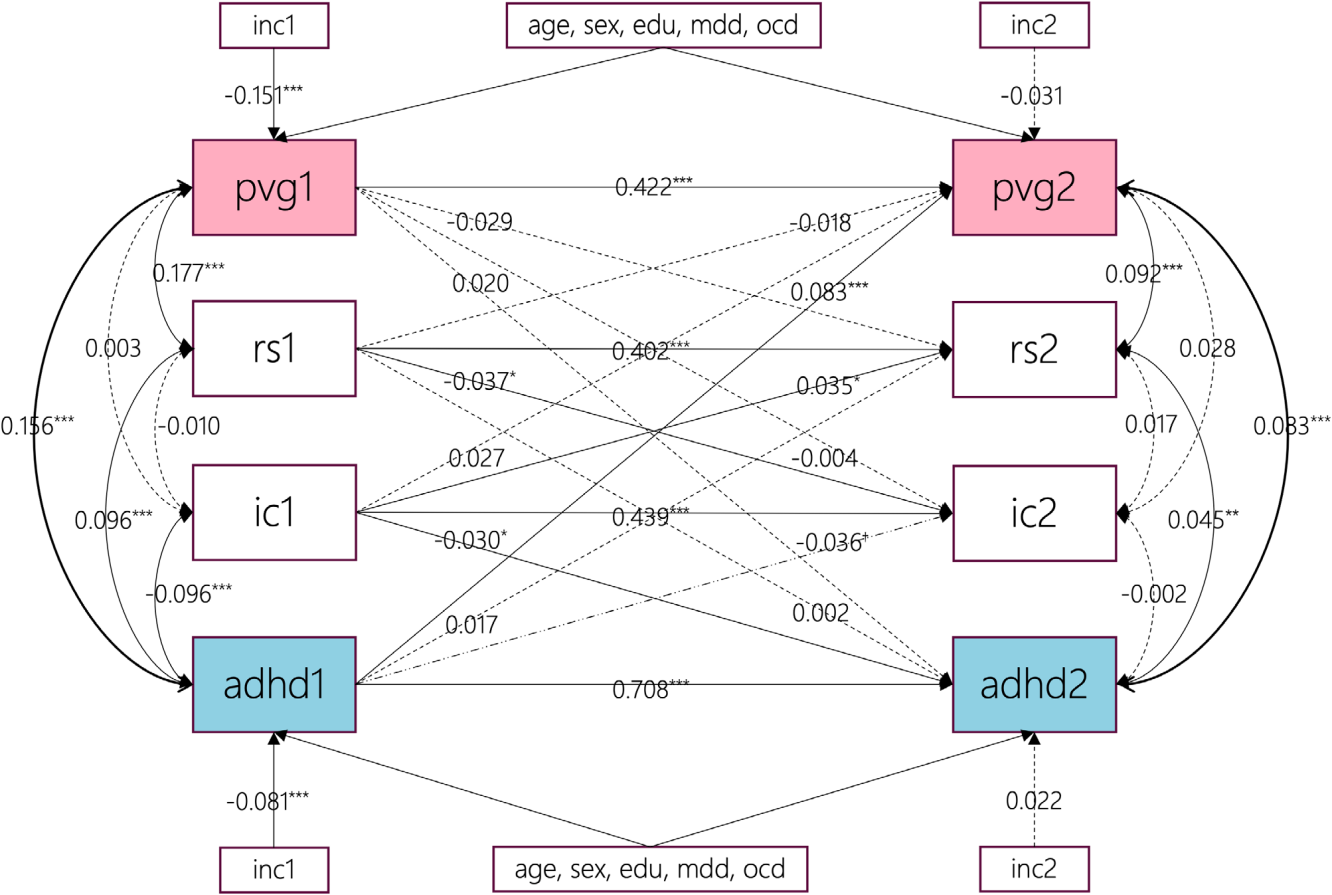
The Cross‐lagged panel model of PVG, ADHD, RS and IC. *p < 0.05, **p < 0.01, ***p < 0.001, † *p* < 0.10; inc: combined family income; edu: primary caregiver's highest educational level; mdd: major depressive disorder; ocd: obsessive‐compulsive disorder. All variables including RS and IC were controlled for age, sex, income, education, MDD, and OCD.

Figure 3 shows the results from the proposed comprehensive CLPM, which indicates that PVG was concurrently significantly associated with RS (*r*_wave1_ = 0.177; *r*_wave2_ = 0.092, *ps* < 0.001) but not with IC. In contrast, ADHD was concurrently associated with RS (*r*_wave1_ = 0.096, *p* < 0.001; *r*_wave2_ = 0.045, *p* = 0.008) and was not consistently related to IC. Regarding the cross‐lagged effects, we found a significant pathway from IC to ADHD (*β* = −0.030, *p* = 0.013) while no significant pathway was found from RS to ADHD (*β* = 0.002, *p* = 0.857). As for the predictors for PVG, neither IC nor RS showed a statistically significant pathway (RS → PVG: *β* = −0.018, *p* = 0.29; IC → PVG: *β* = 0.027, *p* = 0.11). That said, apart from the significant effect of IC predicting subsequent ADHD symptoms, no evidence was found for other directly prospective effects of the dual‐system components on PVG or ADHD. Taken together, H1 was only partially supported. In addition, we found that the lagged effects between RS and IC were significant, where the path coefficients were similar but in opposite directions (RS → IC: *β* = −0.037, *p* = 0.032; IC → RS: *β* = 0.035, *p* = 0.026), suggesting a bidirectional effect between the two systems (i.e., increased inhibitory control may attenuate subsequent reward sensitivity, and vice versa).

### 3.4 Imbalance Degree and Configurations Between RS/IC and PVG/ADHD

To investigate the specific patterns of the interplay between dual systems and PVG/ADHD, RSA was conducted separately for Wave 1 and Wave 2 data (Table 3). The results at Wave 1 were generally consistent with those at Wave 2. RSA results indicated that ADHD severity was associated with both RS (*r*_wave1_ = 0.073, *p* < 0.001; *r*_wave2_ = 0.040, *p =* 0.030) and IC (*r*_wave1_ = −0.070, *p* < 0.001; *r*_wave2_ = −0.045, *p =* 0.041), and the quadratic term of RS was also significantly associated with ADHD severity (*r*_wave1_ = 0.040, *p* < 0.001; *r*_wave2_ = 0.031, *p =* 0.034). In comparison, PVG was only associated with the linear term of RS (*r*_wave1_ = 0.159, *r*_wave2_ = 0.102, *ps* < 0.001). After accounting for the random effects of sites (Table S2), as well as using multiple imputations (Table S3) and full information maximum likelihood (Table S4) to deal with missing data, the findings remained. These findings highlight that the nonlinear relationship of RS with IC may represent an important pattern underlying dual‐system differences between PVG and ADHD.

**TABLE 3:** Polynomial regression and response surface analysis results.

Predictors	PVG		ADHD	
	Wave 1	Wave 2	Wave 1	Wave 2
Sex	−0.340***	−0.114***	−0.148***	−0.262***
Age	0.01	−0.072***	−0.013	0.015
Income	−0.134***	−0.029	−0.078***	−0.107***
Edu	−0.002	0.024	0.037***	0.018
MDD	0.012	0.098***	0.088***	−0.001
OCD	0.051***	0.137***	0.194***	0.015
RS (b1)	0.159***	0.102***	0.073***	0.040*
IC (b2)	−0.007	0.037	−0.070***	−0.045*
RS^2^	0.012	−0.017	0.040***	0.031*
IC^2^	0.003	0.010	0.012	0.015
RS×IC	−0.004	0.001	0.014	0.013
b1−b2	0.166***	0.065*	0.143***	0.085**
	*R* ^2^ = 0.177	*R* ^2^ = 0.090	*R* ^2^ = 0.093	*R* ^2^ = 0.048

*Note:* Wave 1 = 2‐year follow‐up, wave 2 = 4‐year follow‐up.

* *p* < 0.05.

** *p* < 0.01.

*** *p* < 0.001.

**FIGURE 4:**
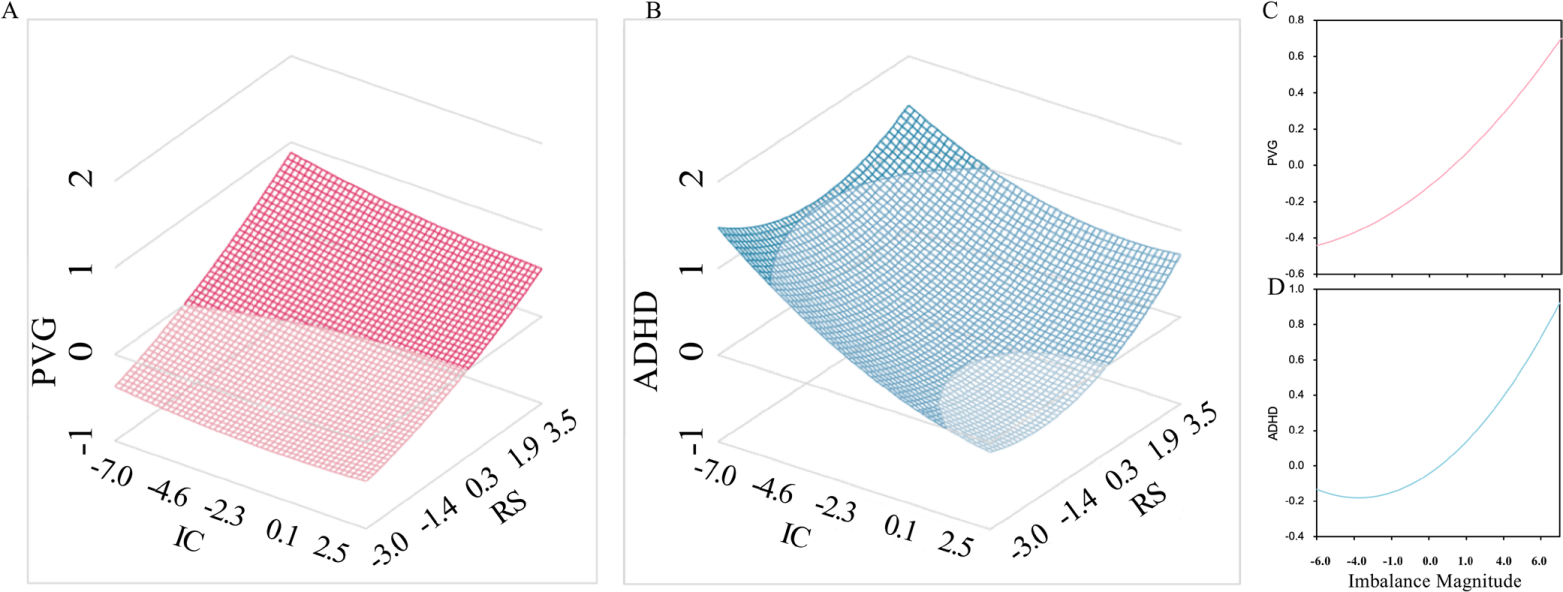
3D and 2D visualization of the association between PVG/ADHD and Dual systems. Visualization was based on Wave 1 (2‐year follow‐up) data. Panel A and B illustrate three‐dimensional relationships between RS, IC, and PVG/ADHD. Panel C and D display incongruent response surfaces, where color lines map PVG/ADHD severity along the imbalance line (RS−IC). As a specific operational example, when RS = 3.0 (hypoactivation), IC = −3.0 (deficiency), the imbalance magnitude equals 6.0.

The response surface coefficients of dual‐system imbalance (b1−b2) on PVG (b1−b2_wave1_ = 0.166, *p* < 0.001, b1−b2_wave2_ = 0.065, *p =* 0.042) and ADHD (b1−b2_wave1_ = 0.143, *p* < 0.001, b1−b2_wave2_ = 0.085, *p =* 0.003) at both waves were significant, indicating that the larger the excess of RS over IC, the more severe the symptoms of PVG and ADHD. Thus, H2 was supported. Figure 4 shows how combinations of IC (*X*‐axis) and RS (*Y*‐axis) map onto predicted PVG/ADHD score (*Z*‐axis). The contrast term *b*_1_ − *b*_2_ indicated how risk changed as RS increased and IC decreased. In Figure 4A,B, this contrast was shown as a surface rising along the diagonal from the region of high IC/low RS to that of low IC/high RS, whereas Figure 4C,D provided a two‐dimensional depiction of this imbalance‐related portion of the surface. Notably, the PVG surface appeared nearly as a plane that increased uniformly along the diagonal direction, whereas the ADHD surface showed a more pronounced pattern of first decreasing and then increasing along the *Y*‐axis. This pattern visually reflected the findings that ADHD, but not PVG, was significantly associated with the quadratic effect of RS (RS^2^). These results suggest that, for PVG, different imbalance configurations with the same overall degree of imbalance may not predict different levels of risk. In contrast, ADHD risk appears to be associated with the specific configuration of imbalance between RS and IC. Taken together, these findings indicate that the imbalance degree is related to both PVG and ADHD, but the particular combinations of RS and IC that confer elevated risk differ between the two behavioral problems.

**FIGURE 5:**
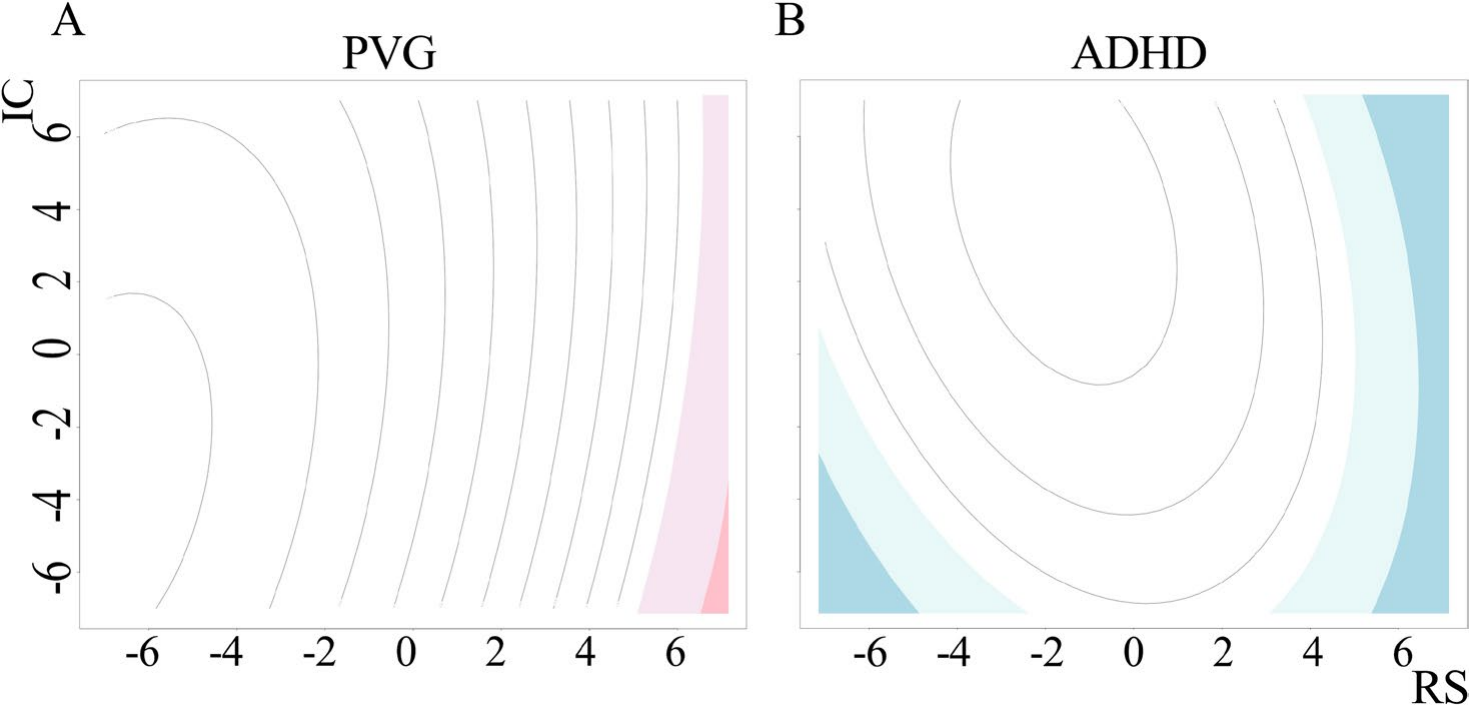
Contour plot of RS and IC combination patterns associated with PVG/ADHD risk states. Risk state classification was based on epidemiological prevalence rates (PVG: Top 10.89% = low‐risk, 6.04% = high‐risk; ADHD: Top 13% = low‐risk, 5.63% = high‐risk) following Meng et al. (2022), CDC criteria (2024), and current sample data. Contour gradients were generated using RSA equations from wave 1 (2‐year follow‐up) data, with color‐coded risk status: Light pink (low‐risk PVG), pink (high‐risk PVG), light blue (low‐risk ADHD), blue (high‐risk ADHD), and white (range with no evidence of risk).

Figure 5 shows the contours of risk states for PVG and ADHD, revealing both shared and specific imbalance configurations in at‐risk status for PVG and ADHD. The color‐shaded regions denote individuals classified as PVG‐ or ADHD‐positive based on previously reported prevalence estimates (Centers for Disease Control and Prevention 2024; Meng et al. 2022) and current sample‐based diagnoses (see Methods and Figure notes for details). The contour lines represent levels of PVG/ADHD symptom severity across combinations of IC and RS. The diagonal extending from low RS/high IC to high RS/low IC reflects increasing degrees of dual‐system imbalance. The nonlinear association between RS and ADHD (RS^2^ term) is manifested in the more pronounced curvature of the contour lines in Figure 5B. For PVG (Panel A), high RS in adolescents, regardless of IC capacity, predisposes them to a higher PVG risk. For ADHD (Panel B), children with elevated RS levels are more likely to develop ADHD; however, even those with low RS, who exhibit diminished IC abilities, also show increased ADHD vulnerability. In summary, PVG and ADHD showed disorder‐specific associations with imbalance configuration based on RS and IC.

## 4 Discussion

PVG and ADHD are intricately linked to RS and IC, yet research lacks detailed exploration of these specific mechanisms (Pfeifer and Allen 2016; Van Den Bos and Eppinger 2016). The CLPM results confirmed the association between PVG/ADHD and RS and IC and provided initial insight into the direction of effects, while RSA detected nuanced linkages between RS, IC, as well as imbalance and PVG/ADHD.

### 4.1 The Complex Longitudinal Interactions Between PVG/ADHD and RS/IC

The dynamic interactions between PVG/ADHD and the RS/IC are more complex than hypothesized (H1). First, we replicated prior reports of the association between PVG and ADHD (Wartberg et al. 2019; Ferguson and Ceranoglu 2014) in the ABCD sample, and our results suggest that ADHD mainly plays a role in driving future PVG and may serve as a vulnerability factor for comorbidity. Second, dual‐system hypotheses of PVG and ADHD were not fully supported. Although lower inhibitory control (IC) is prospectively associated with greater ADHD symptoms after two years, we did not find evidence that RS prospectively predicted PVG and ADHD, or that IC predicted later PVG. Taken together, these cross‐lagged results only partially support H1, that is, when controlling for all proposed confounding variables, only current IC can negatively predict later ADHD. The significant role of IC converges with meta‐analytic evidence documenting persistent inhibitory‐control deficits in ADHD (e.g., Senkowski et al. 2024). In terms of temporal ordering, the effect of IC supports those theoretical models that posit inhibitory‐control deficits as a proximal mechanism of inattention, hyperactivity, and impulsivity (Barkley 1997), where lower IC may serve as an early risk marker for elevated ADHD symptoms.

By contrast, those null cross‐lagged direct effects (i.e., RS → PVG, IC → PVG, RS → ADHD) did not align with what H1 initially proposed and should be interpreted with caution for two reasons. First, neurocognitive developmental processes may have obscured underlying effects. Across adolescence, both reward and inhibitory systems continue to mature, and the discrepancy between the two systems typically narrows over time (Shulman et al. 2016). Such normative developmental changes may attenuate pathology‐related variation, making etiological effects more difficult to detect within a longer‐term developmental context. This interpretation is consistent with the commonly observed phenomenon that PVG symptoms often remit naturally over time. For example, in a one‐year follow‐up study, Wartberg et al. (2019) found that only 14.4% of adolescents with IGD met the diagnostic criteria at both time points, whereas 10.2% met the criteria only at baseline, indicating substantial natural change. Similarly, in a nine‐month follow‐up study of 550 adolescents, Marrero et al. (2021) reported a remission rate of 4.2%, a persistence rate of 2.7%, and a new incidence rate of 6%. In the current dataset, we further found that 6.7% of individuals showed PVG‐positive symptoms at the two‐year follow‐up but not at the four‐year follow‐up. Among individuals who exhibited PVG at least once across the two follow‐ups, this subgroup accounted for 37.1%. Such substantial changes over time may dilute the power of true pathological variation. Second, the 2‐year interval might be too long to capture those effects that operate on shorter timescales. For example, increases in RS triggered by exposure to game‐related cues may lead to transient elevations in PVG symptoms that decay before the next biennial assessment and therefore remain undetected in the current design. Third, according to the dual‐systems framework, it may be the imbalance between reward sensitivity (RS) and inhibitory control (IC), rather than either system in isolation, that plays a more central role in driving maladaptive outcomes. When considered separately, the unique contributions of RS or IC to outcomes such as PVG or ADHD symptoms may be relatively modest, particularly over a long interval such as 2 years. In contrast, theoretical accounts emphasize that risk emerges when heightened reward sensitivity coincides with insufficient inhibitory control. If the joint configuration of these two systems is the critical factor, models focusing only on the independent cross‐lagged effects of RS and IC may underestimate their etiological influence. Fourth, regarding the RS–ADHD relationship, although we did not detect a direct longitudinal effect, the bidirectional relationship between RS and IC may trigger cascading processes linking RS, IC, and ADHD symptoms. However, this inference is limited by the two‐wave design of the current study. Future research with more measurement waves is warranted to test such cascading effects, which may help further refine dual‐system–based theoretical models of ADHD.

### 4.2 Fine‐Grained Patterns of Association Between PVG/ADHD and Dual‐System

Fine‐grained patterns of association between dual systems and PVG/ADHD were revealed through RSA. Our findings showed that ADHD exhibited both a linear correlation with IC and a nonlinear relationship with RS, whereas PVG showed only a linear association with RS. These results were consistent with previous research, where ADHD was associated with both RS (Chen et al. 2024) and IC (Zhang and Li 2022; Zhao et al. 2024), while PVG was only associated with RS (Dong et al. 2022). Nevertheless, the absence of significant correlation between PVG and IC appears inconsistent with some established findings (Sugaya et al. 2019). This discrepancy may be explained by recent ABCD study evidence (Chaarani et al. 2022) suggesting that frequent video gaming enhances cognitive performance, particularly in attentional control during both selection‐based and response‐based processes, which parallels the cognitive demands of the Flanker task (the IC measure in ABCD). Such training effects may potentially obscure expected PVG‐IC relationships. More precise and diverse measurement methods will be needed to clarify the authentic relationship. Regarding nonlinear relationships, we identified a curvilinear association between ADHD and RS, whereas no such pattern was observed with PVG. This finding suggests a potential difference in how these two behavioral outcomes are related to the dual systems. Future research could explore the utility of this mechanism for facilitating the precise classification of PVG and ADHD.

As for the imbalance, overall dual‐system imbalance was positively associated with both PVG and ADHD symptom severity (i.e., greater RS relative to IC was associated with greater risk), providing empirical support for the dual‐systems theoretical framework (Dong and Potenza 2014; Wang and Feng 2023; Wei et al. 2017). However, the configuration of this imbalance differed between the two behavioral problems. This pattern indicates that the degree of imbalance may function as a transdiagnostic risk indicator, whereas differences in the specific configuration of imbalance may help explain divergent symptom manifestations. PVG severity showed a primary association with elevated RS. In contrast, ADHD risk was associated with both elevated RS and reduced IC, as well as the curvilinear effect of RS involvement, indicating that ADHD risk remained elevated once IC fell below a certain point even when RS was low. However, given that the sample was not drawn from clinical settings, these findings can only be informative for generating testable hypotheses for clinical populations. For example, compared with interventions targeting IC, approaches targeting reward processes (e.g., cue‐exposure reduction or alternative reward training) may be particularly promising for PVG. Moreover, interventions simultaneously targeting both RS and IC may yield greater benefits than those focusing on either system alone.

To our knowledge, this study represents an early investigation of the specific mechanisms linking PVG and ADHD through the lens of dual systems. It is also innovative in applying RSA analysis to construct the dual‐system imbalance. The insights offered are multifaceted. First, by directly examining the longitudinal interplay among PVG, ADHD, RS, and IC in a nationally representative sample, this study provides empirical evidence for dual‐system‐based models of ADHD and PVG. Second, the distinct patterns of dual systems observed in PVG and ADHD in this study supplemented the existing dual‐system theoretical models and may offer unique insights into tailored interventions for PVG and ADHD. Third, we provide a novel perspective for assessing imbalance effects and a practical statistical approach to test this perspective using RSA. By empirically evaluating this framework in phenotypes sharing a common dual‐system basis, we uncover intricate relationships between PVG/ADHD, dual systems, and their imbalance. This approach offers a heuristic framework that could facilitate a better understanding of other abnormal behaviors within the context of dual systems models.

### 4.3 Limitations and Future Directions

This study has several limitations that should be considered when interpreting the findings. First, the analysis in this study was based on only two time points, which limits the ability to draw more comprehensive inferences. Longitudinal studies with extended follow‐up are needed to further investigate some of the novel findings that emerged in this study, such as the potential cascading relationships among RS, IC, and ADHD, as well as possible self‐regulatory feedback loops within the dual systems. In addition, this design constrains our ability to identify the optimal time window for detecting effects among the constructs. Future research could benefit from more intensive longitudinal designs that construct and compare multiple sets of lagged parameters within the same dataset (e.g., 1 week, 1 month, 3 months, 6 months, and 12 months) using cross‐lagged models, to identify the time intervals at which these effects are most pronounced. Second, the study used RS and IC as proxies for the dual systems. However, other constructs, such as executive function (including inhibitory control, working memory, and cognitive flexibility), are also considered indicators of the cognitive control system linked with PVG (Soares et al. 2023) and ADHD (Wang and Feng 2023). Further research should encompass a broader range of dual system components. Third, although this study employed a geographically diverse, community‐based sample from the United States, the generalizability of the findings to other cultural contexts and demographic groups remains to be tested, particularly in clinical populations diagnosed with PVG and ADHD. Replication in clinical samples, as well as studies using intervention designs, would help determine whether RS/IC‐based stratification could improve early identification or the tailoring of interventions. Fourth, although the present study employed CLPM and RSA to provide an initial characterization of differences in dual‐system patterns underlying PVG and ADHD, it did not explicitly disentangle within‐person and between‐person sources of variation. Future research would benefit from more intensive longitudinal designs and analytically refined approaches capable of separating these components of variance. For example, studies incorporating three or more closely spaced waves could apply RI‐CLPM to more directly examine within‐person dynamics. Fifth, while the longitudinal design offers evidence regarding temporal ordering among constructs, it does not permit causal inference. To move closer to causal explanation, future work should consider designs and methodologies with stronger causal leverage, such as experimental manipulations and genetically informed approaches. Sixth, although we employed multi‐informant measurement data in the analyses, both PVG and RS were assessed via youth self‐reports. Harman's single‐factor test found that the first factor accounted for about 27% of variance at both time points, which was below the commonly referenced 50% threshold, indicating that a single common factor cannot adequately explain the covariance among items (Tang and Wen 2002). Moreover, prior evidence (Owens et al. 2021) suggests that median within‐reporter correlations among ABCD questionnaire constructs are small (approximately 0.04), further implying limited reporter‐specific inflation. Nevertheless, potential common method bias cannot be entirely ruled out. Therefore, we suggest that the current findings should be interpreted with caution, as the observed association between RS and PVG may be subject to a certain degree of inflation. Seventh, although statistically significant, small effect sizes require careful interpretation. Following the recommendations of (Funder and Ozer 2019), we argue that small effects can nonetheless be meaningful. Even effects of modest magnitude may accumulate through repeated behavioral influences across situations and over time, and in large populations, they can translate into a substantial number of affected cases, which has important implications for public health planning and prevention.

## 5 Conclusion

A complicated longitudinal interplay exists among reward sensitivity (RS), inhibitory control (IC), PVG, and ADHD. Deficient IC predicted subsequent ADHD, and ADHD predicted subsequent PVG. There is a competitive constraining relationship between RS and IC, where IC promotes RS, and RS suppresses subsequent IC. Dual‐system imbalance is the shared mechanism underlying PVG and ADHD, yet distinct imbalance configurations also exist. A larger disparity between RS and IC correlates with more severe symptoms of both PVG and ADHD. While PVG is primarily related to RS, ADHD is related to both RS and IC and exhibits a trend that follows a curved pattern as RS changes. Tailored interventions targeting these specific imbalance patterns may be developed for PVG and ADHD.

## Funding

This work was supported by Brain Science and Brain‐like Intelligence Technology ‐ National Science and Technology Major Project (2021ZD0200500), and National Natural Science Foundation of China, 32571276, 32371142, 32171083, 111 Project, BP0719032.

## Conflicts of Interest

The authors declare no conflicts of interest.

## Supporting information


**Data S1:** pchj70113‐sup‐0001‐supinfo.docx.
**Table S1:** Original and Re‐labeled Time Labels and Interview Date Ranges.
**Table S2:** Coefficients After Accounting for Site‐Level Random Effects.
**Table S3:** Coefficients From RSA Analysis Using Multiple Imputation.
**Table S4:** Coefficients From RSA Analysis Using FIML.

## Data Availability

The code that support the findings of this study are available on request from the corresponding author. The data are not publicly available due to privacy or ethical restrictions.
