# Supplementary material for: Shared and Unique Patterns of Dysregulated Dual Systems Between Adolescent Problematic Video Gaming and ADHD
Source: Psych J. 2026 Jul 13;15(4):e70113. doi: 10.1002/pchj.70113 (PMC13365367; doi:10.1002/pchj.70113)
Supplement: Supplementary file 1 — Data S1: pchj70113‐sup‐0001‐supinfo.docx. Table S1: Original and Re‐labeled Time Labels and Interview Date Ranges. Table S2: Coefficients After Accounting for Site‐Level Random Effects. Table S3: Coefficients From RSA Analysis Using Multiple Imputation. Table S4: Coefficients From RSA Analysis Using FIML. [file PCHJ-15-e70113-s001.docx]

**Supplementary Materials**

for

Shared and Unique Patterns of Dysregulated Dual Systems between Adolescent Problematic Video Gaming and ADHD

**Table S1**

*Original and Re-labeled Time Labels and Interview Date Ranges*

| Original time label | Re-labeled time label | Interview time |
| --- | --- | --- |
| 2_year_follow_up_y_arm_1 | wave 1 | 2018-2021 |
| 4_year_follow_up_y_arm_1 | wave 2 | 2020-2022 |

**Supplement 2. Mathematical Derivation Demonstrating That b₁ − b₂ Captures Dual-System Imbalance**

Let x_1_=RS，x_2_=IC.

Define the *mean* M and *difference* D (our imbalance index) as

M=$\frac{x_{1}+x_{2}}{2}$, D=$x_{1}$-$x_{2}$.

Then

x_1_=M+$\frac{1}{2}$D, x_2_=M-$\frac{1}{2}$D.

The typical RSA model we fit is:

$$y_{i}=b_{0}+{b_{1}x}_{1i}+b_{2}x_{2i}+b_{3}x_{1i}^{2}+{b_{4}x_{1i}x_{2i}+b}_{5}x_{2i}^{2}+e_{i}$$

Substituting x_1_ and x_2_ in terms of M and D and simplifying yields:

$$y_{i}=b_{0}+(b_{1}+b_{2})M+\frac{b_{1}-b_{2}}{2}D+(b_{3}+b_{4}+b_{5})M^{2}+(b_{3}-b_{5})MD+\frac{(b_{3}-b_{4}+b_{5})}{4}D^{2}+e_{i}$$

The linear coefficient on D is $\frac{b_{1}-b_{2}}{2}$​​. Thus, a significant b_1_−b_2_ indicates a *linear imbalance effect* (i.e., higher RS relative to IC — or vice versa — linearly relates to outcome differences).

**Supplement** **3. Details for Covariate Adjustments**

Crucial demographic variables, including child sex, age, race, combined family income, and the primary caregiver's highest educational attainment (derived by taking the maximum value between paternal and maternal educational levels, which were originally measured separately), were treated as covariates since they are commonly regarded as having a significant relationship with the predictor and outcome variables. Additionally, Major Depressive Disorder (MDD) and Obsessive-Compulsive Disorder (OCD), shared comorbidities of PVG and ADHD (DSM-5-TR, 2022), were also controlled as covariates, and due to insufficient time points for measurement, the value of the baseline is used as the covariate for the two-year follow-up and four-year follow-up in a time-invariant manner.

Aligned with previous studies utilizing the ABCD data, we have included a set of demographics and psychiatric confounding factors as covariates in the Cross-Lagged Panel Model (CLPM) and Response Surface Analysis (RSA). For the Problematic Video Gaming (PVG) data analyses, sex, age, race, combined family income, the highest education attainment of the caregiver, as well as MDD and OCD were found to be associated with PVG (Woodman, 2023; Chaarani et al., 2022). These demographic and psychiatric variables have also been reported to be correlated with children’s mental health (CBCL) (Barch et al., 2021), neurocognitive performance (NIH toolbox cognitive battery) (Thompson et al., 2019; Pagliaccio et al., 2020), and reward sensitivity (RS) (McNeilly et al., 2022; Freeman et al., 2023). Given that most of these measurements are correlated with children’s PVG, dual systems, and ADHD, they may induce potential confounding bias in the estimated relationships between predictor and outcome variables (Hamaker et al., 2020). Thus, we decided to include these covariates in CLPM and RSA, respectively.

**Table S2**

*Coefficients After Accounting for Site-Level Random Effects*

|  | PVG (2-year) | PVG (4-year) | ADHD (2-year) | ADHD (4-year) |
| --- | --- | --- | --- | --- |
| Variable | Estimate | Estimate | Estimate | Estimate |
|  | R^2^ = 0.177 | R^2^ = 0.090 | R^2^ = 0.093 | R^2^ = 0.048 |
| RS(b1) | 0.157^***^ | 0.095^***.^ | 0.071^***^ | 0.041^*^ |
| IC(b2) | -0.005 | 0.043 | -0.070^***^ | -0.044^*^ |
| RS^2^ | 0.011 | -0.018 | 0.040^***.^ | 0.029* |
| $RS\times IC$ | 0.003 | 0.000 | 0.014 | 0.012 |
| IC^2^ | 0.001 | 0.013 | 0.012 | 0.015 |
|  |  |  |  |  |
| b1-b2 | 0.162^***^ | 0.052^*^ | 0.141^***^ | 0.085^**^ |

*Note*. Unstandardized coefficients were estimated using multilevel response surface analysis (mRSA; Nestler et al., 2019).

**Table S****3**

*Coefficients From RSA Analysis Using Multiple Imputation*

|  | PVG (wave2) | PVG (wave4) | ADHD (wave2) | ADHD (wave4) |
| --- | --- | --- | --- | --- |
| Predictors | Estimate | Estimate | Estimate | Estimate |
| RS(b1) | 0.112^***^ | 0.082^***^ | 0.079^***^ | 0.041^***^ |
| IC(b2) | -0.013 | 0.006 | -0.056^***^ | -0.040^**^ |
| RS^2^ | 0.020^***^ | -0.007^***^ | 0.039^***^ | 0.041^***^ |
| $RS\times IC$ | -0.012^**^ | 0.002 | -0.001 | -0.003 |
| IC^2^ | 0.002 | 0.017 | 0.002 | 0.003 |
|  |  |  |  |  |
| $b1-b2$ | 0.125^***^ | 0.075^***^ | 0.135^***^ | 0.081^***^ |

*Note*. Multiple imputation was performed using the `mice` package in R to generate 100 imputed datasets. Each dataset was analyzed using response surface analysis (RSA), and the final coefficients represent the mean estimates across the 100 RSA models. Wave 1 = 2-year follow-up; Wave 2 = 4-year follow-up.

**Table S4**

*Coefficients From RSA Analysis Using FIML*

|  | PVG | | ADHD | |
| --- | --- | --- | --- | --- |
| Predictors | Wave 1 | Wave 2 | Wave 1 | Wave 2 |
| RS ($b1$) | 0.159^***^ | 0.102^***^ | 0.073^***^ | 0.040^*^ |
| IC ($b2$) | -0.007 | 0.037 | -0.070^***^ | -0.045^*^ |
| RS^2^ | 0.012 | -0.017 | 0.040^***^ | 0.031^*^ |
| IC^2^ | 0.003 | 0.010 | 0.012 | 0.015 |
| $RS\times IC$ | -0.004 | 0.001 | 0.014 | 0.013 |

*Note*. Coefficients were estimated using the `lavaan` package in R, with missing data handled using full information maximum likelihood (FIML).

**References**

American Psychiatric Association. (2022). *Diagnostic and Statistical Manual of Mental Disorders*, Fifth Edition, Text Revision (DSM-5-TR). Washington, DC: American Psychiatric Association.

Barch, D. M., Albaugh, M. D., Avenevoli, S., Chang, L., Clark, D. B., Glantz, M. D., Hudziak, J. J., Jernigan, T. L., Tapert, S. F., Yurgelun-Todd, D., Alia-Klein, N., Potter, A. S., Paulus, M. P., Prouty, D., Zucker, R. A., & Sher, K. J. (2018). Demographic, physical and mental health assessments in the adolescent brain and cognitive development study: Rationale and description. *Developmental Cognitive Neuroscience*, *32*, 55–66. <https://doi.org/10.1016/j.dcn.2017.10.010>

Chaarani, B., Ortigara, J., Yuan, D., Loso, H., Potter, A., & Garavan, H. P. (2022). Association of video gaming with cognitive performance among children. JAMA network open, 5(10), e2235721-e2235721.

Freeman, C., Olino, T., Barbeau, E. B., Weinberg, A., & Chai, X. (2023). Family history of depression and neural reward sensitivity: Findings from the Adolescent Brain Cognitive Development Study. Biological Psychiatry: Cognitive Neuroscience and Neuroimaging, 8(6), 620-629.

Hamaker EL, Mulder JD, van IMH, (2020). Description, prediction, and causation: Methodological challenges of studying child and adolescent development. Dev Cogn Neurosci. 46:100867.

McNeilly, E. A., Saragosa-Harris, N. M., Mills, K. L., Dahl, R. E., & Magis-Weinberg, L. (2022). Reward sensitivity and internalizing symptoms during the transition to puberty: An examination of 9-and 10-year-olds in the ABCD Study. Developmental Cognitive Neuroscience, 58, 101172.

Pagliaccio, D., Alqueza, K. L., Marsh, R., & Auerbach, R. P. (2020). Brain volume abnormalities in youth at high risk for depression: adolescent brain and cognitive development study. Journal of the American Academy of Child & Adolescent Psychiatry, 59(10), 1178-1188.

Thompson WK, Barch DM, Bjork JM, et al, (2019). The structure of cognition in 9- and 10-year-old children and associations with problem behaviors: Findings from the ABCD study's baseline neurocognitive battery. Dev Cogn Neurosci. 36:100606.

Woodman, K. S. C. (2023). Neurobiological Predispositions and Developmental Trajectories of Gaming Disorder in Adolescents: A Longitudinal ABCD Study Analysis (Doctoral dissertation, UC Santa Barbara).
